# Manufacturing Parameters for the Creation of Clinical-Grade Human-Induced Pluripotent Stem Cell Lines From Umbilical Cord Mesenchymal Stromal Cells

**DOI:** 10.1093/stcltm/szae010

**Published:** 2024-02-25

**Authors:** Liziane Raquel Beckenkamp, Camila Gomes da Silva, Mônica Luiza Immig Von Hohendorff, Karolyn Sassi Ogliari

**Affiliations:** Hemocord Biotecnologia, Tecnosinos—Technologic Park, São Leopoldo, RS, Brazil; Hemocord Biotecnologia, Tecnosinos—Technologic Park, São Leopoldo, RS, Brazil; Hemocord Biotecnologia, Tecnosinos—Technologic Park, São Leopoldo, RS, Brazil; Hemocord Biotecnologia, Tecnosinos—Technologic Park, São Leopoldo, RS, Brazil

**Keywords:** induced pluripotent stem cells, umbilical cord tissue, mesenchymal stem cells, cellular therapy, clinical translation

## Abstract

Induced pluripotent stem cells (iPSCs) are reprogrammed cells with a remarkable capacity for unlimited expansion and differentiation into various cell types. Companies worldwide are actively engaged in developing clinical-grade iPSC lines to address the needs of regenerative medicine, immunotherapies, and precision medicine. However, ensuring the safety and quality of iPSCs is essential, with adherence to Good Manufacturing Practices (GMP) and ethical considerations being paramount. Perinatal cell and tissue banks, such as umbilical cord (UC) blood and tissue banks, are emerging as ideal sources for generating iPSCs due to their unique characteristics and GMP compliance. These banks provide access to immature cells with limited environmental exposure, known family and medical histories of donors, and readily available resources, thereby reducing the time and cost associated with personalized treatment strategies. This study describes the establishment of the first clinical-grade iPSC lines from umbilical cord mesenchymal stromal cells in Brazil. The process involved rigorous quality control measures, safety assessments, and adherence to regulatory standards, resulting in iPSCs with the necessary characteristics for clinical use, including sterility, genomic integrity, and stability. Importantly, the study contributes to the development of a Current Good Manufacturing Practice-compliant iPSC production pipeline in Brazil, using commercially available, chemically defined, and xeno-free products, along with validation by national outsourced laboratories, thereby facilitating the adoption of this technology within the country. The study emphasizes Brazil’s contribution to the progress of translational medicine and the promotion of scientific advancements within the field of regenerative and precision medicine.

## Graphical Abstract

**Graphical Abstract:**
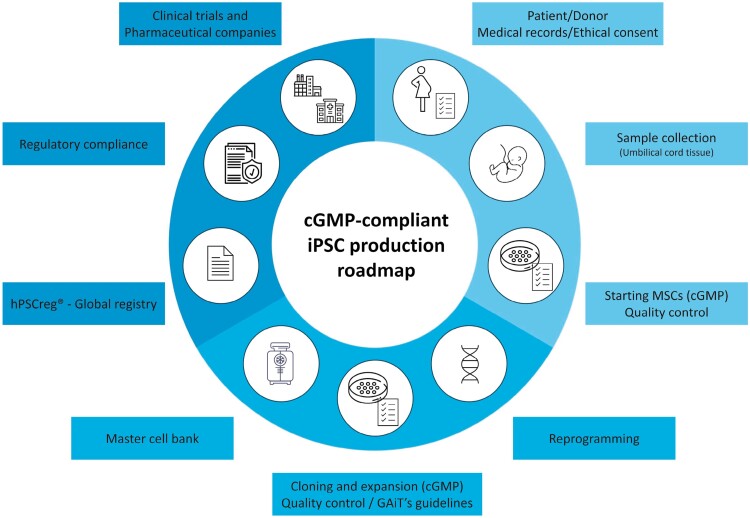

**Significance Statement**

This study highlights the groundbreaking advancements in induced pluripotent stem cell (iPSC) technology in Latin America, specifically in Brazil. It underscores its importance for regenerative medicine and translational iPSC applications. The research prioritizes stringent quality control and safety measures, adhering to Good Manufacturing Practices, which can benefit clinical researchers globally. Addressing concerns about genomic stability and safety, the study contributes to essential discussions in the field. The commitment to global collaboration and standardization promotes the accessibility of this technology worldwide, propelling scientific progress.

## Introduction

Induced pluripotent stem cells (iPSCs) are reprogrammed somatic cells that possess the unique ability to revert to a pluripotent state, enabling them to have the potential for unlimited expansion and differentiation into various cell types.^1^ This makes them highly promising for applications in regenerative medicine, disease modeling, precision medicine, drug testing, and pharmacogenomics.^2^ Currently, more than 40 commercial companies around the world are developing iPSC lines for clinical purposes and clinical trials are in progress to investigate the use of iPSC-derived cells for regenerative treatments in cardiac, neural, metabolic, and eyes diseases, as well as for immune diseases and cancer.^3^

However, the clinical translation of iPSCs comes with challenges. Ensuring the safety and quality of iPSCs is imperative and strict quality control (QC) measures and adherence to Good Manufacturing Practices (GMP) are necessary to meet regulatory standards for clinical use.^4,5^ In 2018, the Global Alliance for iPSC Therapies (GAiT) established guidelines for the QC of clinical-grade iPSC lines, with the aim of advancing iPSC-based treatments toward clinical reality while prioritizing safety, efficacy, and patient access.^6^ Additionally, ethical considerations regarding the source of somatic cells, patient consent, and data privacy must be carefully addressed.^7^

In this context, perinatal cell and tissue banks, such as umbilical cord (UC) blood and tissue banks, are particularly highlighted as excellent sources for generating iPSCs because they store immature cells that have had minimal exposure to environmental factors and possess comprehensive family and medical records, offering a personalized and readily available source for regenerative medicine. Moreover, these banks operate in GMP-compliant facilities and have well-established QC processes, ensuring the safety of iPSCs for clinical use.^8-10^ Thus, these cell processing centers could offer patient-specific iPSCs platforms for studying disease mechanisms and screening potential drugs, potentially reducing the time and resources required to develop personalized treatment strategies,^11,12^ as well as cell products derived from patient-specific iPSCs to treat numerous diseases with a lower risk of immune rejection, thereby reducing waiting times for transplants.^3^

Here, we describe the establishment of the first clinical-grade iPSC cell lines from umbilical cord mesenchymal stromal cells (UC-MSCs) in Brazil, with a specific emphasis on creating a pipeline using commercially available reagents manufactured in compliance with Current Good Manufacturing Practice (cGMP) requirements, while guaranteeing a thorough safety assessment of the cells.

## Materials and Methods

The generation and characterization of these iPSC lines were approved by the Human Research Ethics Committee of the University of Vale do Rio dos Sinos (Unisinos; no 09.02.2021). Umbilical cord tissue was donated by pregnant women who hired Hemocord Biotecnologia’s service to collect UC blood, after signing an informed consent.

The UC was processed in a GMP-compliant facility. The tissue was digested with the Umbilical Cord Dissociation kit (Miltenyi Biotec), following the manufacturer’s instructions, and UC-MSCs were cultured under xeno-free conditions up to passage 3 (Table 1). The P0 (master cell bank) and P3 UC-MSCs were tested for QC release for cell viability, sterility, mycoplasma, endotoxins, viral diseases, genetic stability, as well as for phenotypic characterization of the MSCs by flow cytometry and functional assays, such as cell differentiation in mesodermal lineages and lymphocyte proliferation assay. All analyses were carried out by certified outsourced laboratories.

**Table 1.** Media, kits, and solutions used to manufacture clinical-grade iPSCs.

Procedure	Product	CTS	cGMP manufactured	Xeno-free	AOF	Company	Catalog number
UC-MSCs expansion	Umbilical Cord Dissociation Kit					Miltenyi Biotec	130105737
	CTS DMEM/F-12 Knockout	✓	✓		✓	Gibco	A1370801
	PLUS GMP grade human platelet lysate		✓	✓		Compass Biomedical	PLSGB-0100
	CTS DPBS	✓	✓		✓	Gibco	A12856
	CTS TrypLE Express Enzyme		✓		✓	Gibco	12604
	Penicillin-Streptomycin		✓			Gibco	15140122
Reprogramming	CTS CytoTune-iPS 2.1 Sendai Reprogramming Kit	✓	✓		✓	Invitrogen	A34546
	CTS DMEM/F-12 Knockout	✓	✓		✓	Gibco	A1370801
	CTS GlutaMAX-I Supplement	✓	✓		✓	Gibco	A1286001
	CTS SR XenoFree Knockout	✓	✓	✓		Gibco	12618012
	Recombinant human fibrolblast growth factor-basic (FGF-b)				✓	Gibco	PHG0024
	CTS Vitronectin (VTN-N) Recombinant Human Protein	✓	✓		✓	Gibco	CTS279S3
	CTS Essential 8 Medium	✓	✓		✓	Gibco	A2656101
	Penicillin-Streptomycin		✓			Gibco	15140122
iPSC expansion	CTS Vitronectin (VTN-N) Recombinant Human Protein	✓	✓		✓	Gibco	CTS279S3
	CTS RevitaCell Supplement	✓	✓		✓	Gibco	A4238401
	CTS Essential 8 Medium	✓	✓		✓	Gibco	A2656101
	Penicillin-Streptomycin		✓			Gibco	15140122
	Recombinant human FGF-b				✓	Gibco	PHG0024
	CTS DPBS	✓	✓		✓	Gibco	A12856
	CTS Versene Solution	✓	✓		✓	Gibco	A4239101
iPSC cryopreservation	CTS Synth-a-Freeze Medium	✓			✓	Gibco	A1371301
Embryoid body	CTS DMEM/F-12 Knockout	✓	✓		✓	Gibco	A1370801
	CTS GlutaMAX-I Supplement	✓	✓		✓	Gibco	A1286001
	CTS SR XenoFree knockout	✓	✓	✓		Gibco	12618012
	Penicillin-Streptomycin		✓			Gibco	15140122
	CTS RevitaCell Supplement	✓	✓		✓	Gibco	A4238401

Abbreviations: CTS, Cell Therapy Systems; cGMP, Current Good Manufacturing Practice; AOF, animal origin-free; ✓, accordingly.

Reprogramming was performed using the Cell Therapy Systems (CTS) CytoTune-iPS 2.1 Sendai Kit, following the manufacturer’s instructions. Colonies exhibiting iPSC-like morphology were manually selected 3 weeks post-transduction and were maintained on CTS Vitronectin. The clones were grown on CTS Essential 8 medium plus 10 ng/mL FGF, with daily medium changes, and cells were split every 5-7 days using CTS Versene solution. iPSC lines were cultivated in GMP conditions (Table 1) until at least passage 10 before characterization, which was carried out following the recommendations of the GAiT on the minimum set of data required to be considered a clinical-grade iPSC line.^6^ All analyses were carried out by certified outsourced laboratories using the analytical methodologies described in Supplementary Table S1. Potency assay for the formation of embryoid bodies (EBs) and differentiation into the 3 germ cells was carried out in-house (Supplementary Table S1).

## Results

Before the establishment of the clinical-grade iPSC lineages from UC-MSCs, some critical elements were fulfilled. The consent provided by the participant included that the iPSCs created from their biological material, as well as the information from their medical records, could be stored indefinitely. The genetic information originated from the samples could be collected, stored, and made available for future research, and they could be contacted regarding the relevant results. The participant was made aware that genetic testing may generate unexpected findings unrelated to the primary reason for studing (secondary findings), and there is a possibility that the significance of certain genetic findings may change over time (eg, if a variant of uncertain significance is later reclassified as pathogenic several years later), and that they may not be notified. The participant has also consented to these cells being made available to public and private institutions, for both profit and not-for-profit, for use in basic, preclinical, and clinical research around the world, protecting the confidentiality of their name, and in these cases, the results of the analyses conducted may not be communicated to them. They recognize that the insights derived from these cells may lead to the development of new patents and commercial products based on the techniques developed. However, it is made clear that no commercial products will be derived directly from their cells and, consequently, they will not receive any financial compensation in the future. After consent, a rigorous donor screening was carried out before UC tissue collection, involving medical records and clinical laboratory testing, which were negative for relevant communicable disease agents or diseases.

**Figure 1.**
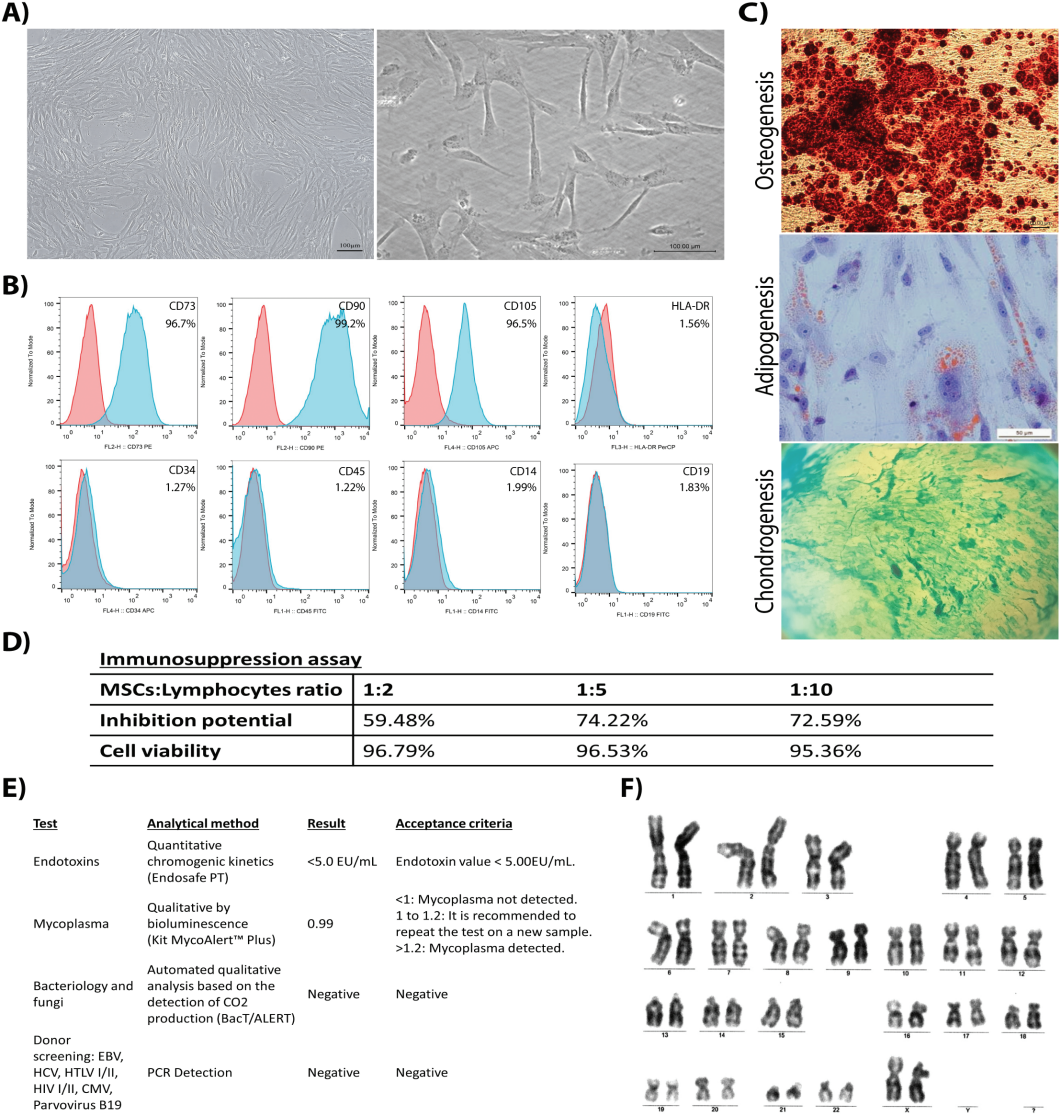
Characterization of UC-MSCs. (**A**) Representative phase-contrast photomicrographs of cultured UC-MSCs showing spindle-shaped morphology. Scale bar = 100 μm. (**B**) Flow cytometry results for MSCs-specific markers (CD73, CD90, and CD105) and negative for CD34, CD45, CD14, CD19, and HLA-DR. (**C**) UC-MSCs differentiated in the 3 mesodermal lineages: osteogenic (Alizarin Red S staining—scale bar = 100 μm), adipogenic (Oil Red O staining—scale bar = 50 μm), and chondrogenic (Alcian blue staining—scale bar = 50 μm). (**D**) Immunosuppression potential of UC-MSCs in coculture with human lymphocytes. (**E**) Sterility results and viral screening. (**F**) G-band staining showing UC-MSCs normal karyotype.

UC-MSCs were isolated, expanded, and characterized in passage 3 according to local regulatory requirements^13^ and the International Society for Cellular Therapy’s criteria defining MSCs.^14^ These cells met all the required criteria, such as fibroblast morphology, expression of MSCs markers, multilineage differentiation, immunosuppressive potential, as well as sterility requirements, viral tests, and genomic stability (Figure 1).

The reprogramming of UC-MSCs had a transduction efficiency of approximately 0.01%. In total, 19 iPSC lines were manually selected, 10 of them survived and actively proliferated in culture after the colony isolation step. The clones have been identified using the standard nomenclature established by hPSCreg, a global registry of iPSC lines with the aim of certifying the quality and ethical provenance of iPSC lines, as well as increasing the visibility and availability of these cells developed around the world.^15^

**Figure 2.**
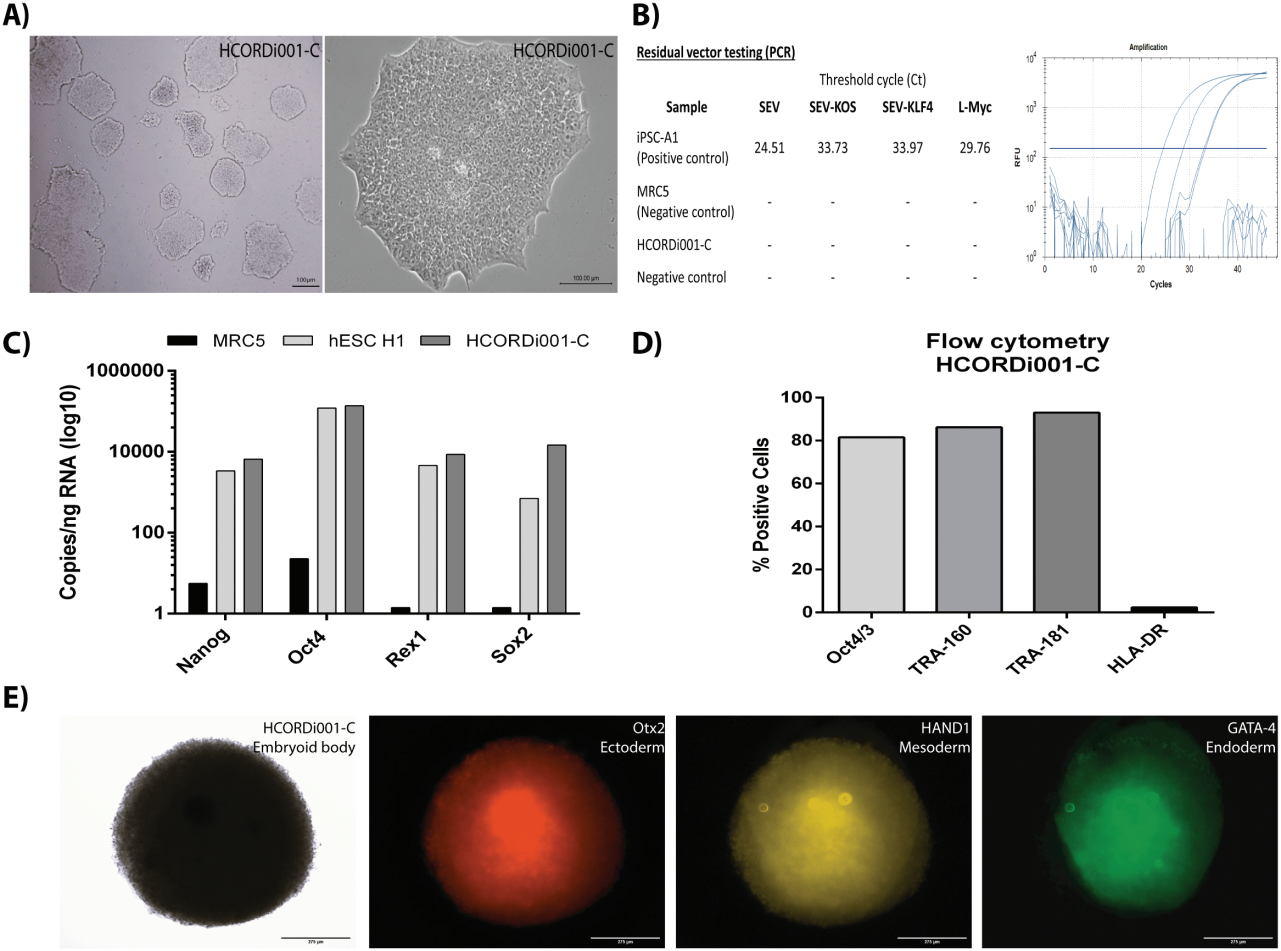
Characterization of the HCORDi001-C iPSC line. (**A**) Morphology of iPSC colonies at passage 12 (scale bar = 100 μm). (**B**) Detection of residual reprogramming vector by qPCR. Controls: iPSC-A1 as positive control and MRC5 (fibroblasts) as negative control. (**C**) Absolute quantification of pluripotency gene expression by droplet PCR digital (ddPCR). MRC5 (fibroblasts) as negative control and hESC H1 (embryonic cells) as positive control. (**D**) Stemness markers were used to evaluate iPSC by flow cytometry. (**E**) Immunofluorescent staining for ectoderm marker Otx2, mesoderm HAND1, and endoderm GATA4. Scale bar = 275 μm.

To develop clinical-grade iPSCs, we used CTS products, which have been validated for the generation of clinical iPSCs by different research groups around the world.^16^ All iPSC lines exhibited a typical embryonic stem cell-like morphology, high nucleus-to-cytoplasm ratios, and prominent nucleoli (Fig. 2A; Supplementary Fig. S1A). Gene expression analysis using Sendai virus-specific primers confirmed that the vectors were eliminated from passage 10 (Fig. 2B). However, HCORDi001-A, HCORDi001-B, HCORDi001-E, and HCORDi001-H iPSC lines needed to undergo a temperature shift to remove KOS and L-Myc vectors, as recommended by the manufacturer of the CTS CytoTune iPSC 2.1 Sendai reprogramming kit, before the cells could be characterized.

The pluripotency of iPSC was assessed by gene expression (ddPCR), which showed the presence of the pluripotency markers Oct4 (Pou5f1), Sox2, Nanog, and Rex1, at levels similar to or higher than human embryonic stem cell line (hESC H1; Fig. 2C; Supplementary Fig. S1B). Flow cytometry analyses also showed the expression of the widely accepted stemness markers TRA-1-81, TRA-1-60, and OCT3/4. Of the iPSC lines evaluated, 4 showed more than 70% of the cells positive for all pluripotency markers and 5 showed at least 50% of cells positive for all markers. Furthermore, we evaluated the expression of the histocompatibility marker MHC-II (HLA-DR), showing that expression was lower than 2% in all iPSC lines (Fig. 2D; Supplementary Fig. S1C). Lastly, we demonstrated that all iPSCs lines successfully generated EBs and immunohistochemistry confirmed the expression of markers denoting each germ layer: Otx2 (ectoderm), HAND1 (mesoderm), and GATA4 (endoderm; Fig. 2E; Supplementary Fig. S1D).

**Figure 3.**
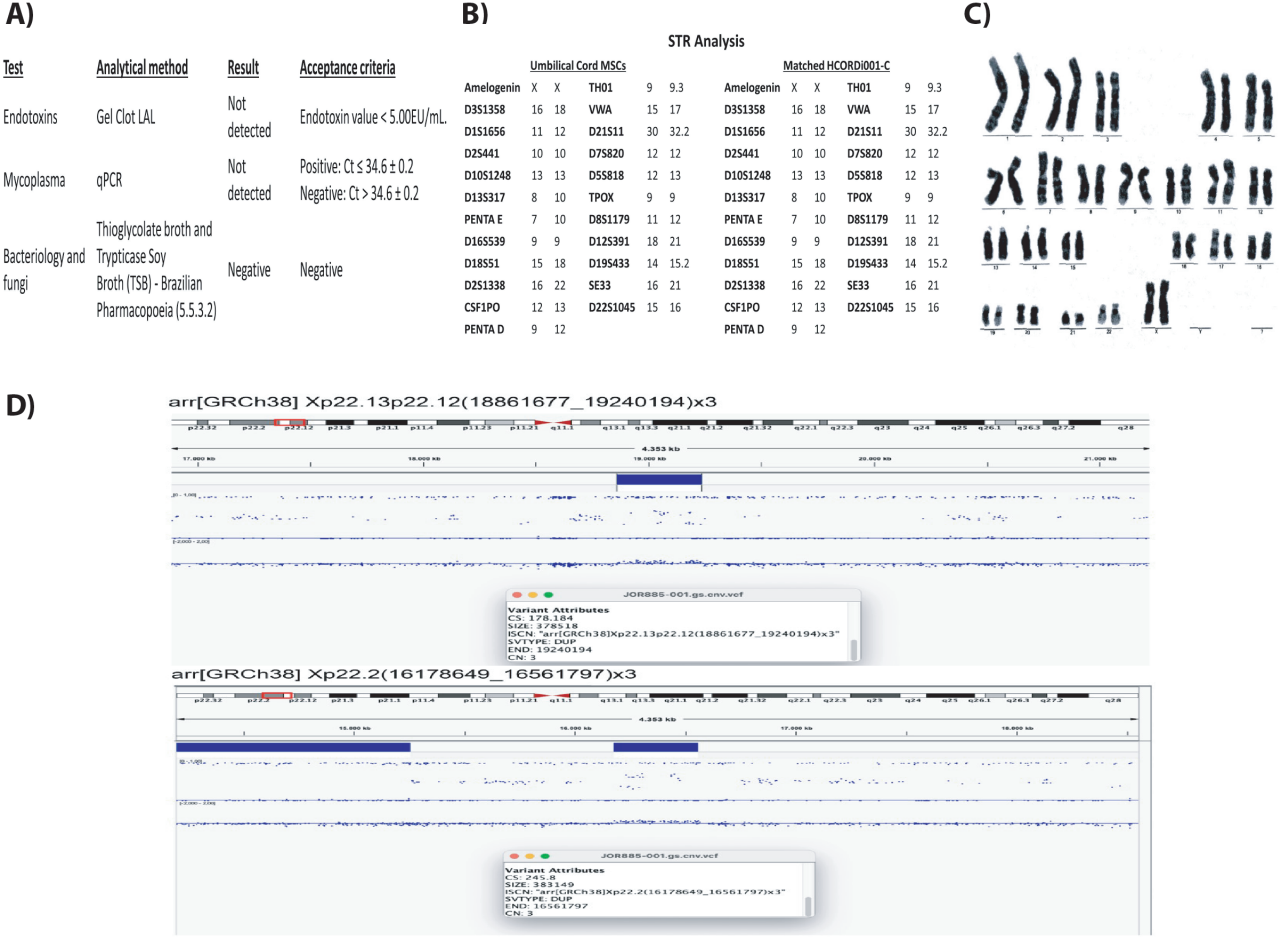
Safety assessment of the HCORDi001-C iPSC line. (**A**) Sterility results. (**B**) STR analysis showing that the iPSCs matched the UC-MSCs donor sample. (**C**) iPSCs demonstrating normal karyotype after 12 passages. (**D**) Variants with no clinical significance were detected by SNP array analysis.

To evaluate whether the clinical-grade iPSCs lines were biologically safe, we performed a series of safety assays. All iPSC lines were sterile, with negative results for mycoplasma, endotoxins, bacteria, and fungi (Fig. 3A; Supplementary Fig. S2A). Cell identity was confirmed by short tandem repeat analysis (STR) compared to starting UC-MSCs (Fig. 3B; Supplementary Fig. S2A), and the karyotype detected by G-band analysis demonstrated that the iPSCs lines carried a normal diploid karyotype (Fig. 3C; Supplementary Fig. S2B).

To obtain a more robust information on chromosomal alterations, we performed single nucleotide polymorphism (SNP) array analyses using the GRCh38 version of the human genome as a reference. From a clinical point of view, the analyses did not detect any gain or loss of chromosomal segments considered pathogenic, probably pathogenic, or of uncertain clinical significance in the iPSC lines. However, the results showed that UC-MSCs had 2 nonpathogenic variants that were maintained in the iPSC lineages, with the start or end of microduplication at different points. Furthermore, HCORDi001-E and HCORDi001-J iPSC lines showed a nonpathogenic microduplication that was not detected in the UC-MSCs, while clone HCORDi001-I showed a nonpathogenic microdeletion (Fig. 3D; Supplementary Fig. S2A). The average percentage of these nonpathogenic variants in the iPSC lines was 0.112% ± 0.01%. Finally, we performed a genomic analysis by next generation sequencing for an expanded cancer panel containing 264 cancer-associated genes, which was customized by Illumina company to encompass all exons of the human genome, including exon-intron borders, as well as the mitochondrial genome (Supplementary Table S2). No pathogenic variants or variations in the number of copies (CNVs) were detected in genomic regions known to be associated with the development of cancer (data not shown). However, 3 variants of uncertain significance (VUS) were observed in all iPSCs, which were inherited from the parental lineage (UC-MSCs): CHEK2 (variant: 22-28725372-A-T), MYCN (variant: 2-15940575-C-T), and PARN (variant: 16-14555675-G-T).

## Discussion

Since its discovery in 2006,^17^ iPSC technology has rapidly evolved and has become an important tool in the development of new therapeutic drugs, disease modeling, and potential treatment of various diseases. Results from the first clinical trials involving iPSCs are beginning to emerge, showing promising outcomes in the treatment of conditions such as age-related macular degeneration,^18^ platelet transfusion,^19^ graft-versus-host disease, ^20^ and type 1 diabetes.^21,22^

Ensuring safety and reproducibility in the production of clinical-grade iPSCs is essential for the success of these therapies. In recent years, we have improved our facilities and quality framework, obtaining certification from the Association for the Advancement of Blood & Biotherapies and biosafety quality certificate, and fulfilled other legal requirements to initiate the development of clinical-grade iPSCs.^23^ Results of this study reflect the effectiveness of these actions, demonstrating the creation of various iPSC lines that meet the requirements for clinical use, including sterility, identity tests, potency, and safety.

One of the main concerns with the development of iPSCs is their genomic stability and whether genetic variations could increase disease risk when iPSC-derived cells/tissues are used in the clinic.^24^ Our genetic analyses of the iPSCs revealed the absence of chromosomal alterations or CNVs. Most notably, we did not identify any pathogenic or potentially pathogenic variants, or mutations in cancer-associated genes, in the samples evaluated. Nonpathogenic variants identified in our SNP array revealed that most of them were preexisting in the parental cells. Only 3 iPSC lines showed variants that may have been acquired during reprogramming or early culture of the iPSC lines, and these were detected at low frequency. Since there is no concrete information on whether they are likely to be pathogenic or benign, this does not make their use unfeasible so far. Similarly, the VUS identified in the hereditary cancer panel analyses were also present in the parental cells. Studies have suggested that a more comprehensive genetic analysis of the somatic cells of origin should be conducted before reprogramming, and a clonal selection of them could potentially reduce the incidence of observed genetic variants.^25^

Another important issue is the immunogenicity of iPSCs. To address this problem, researchers around the world are developing iPSC biobanks containing homozygous cell lines to allow HLA matching for a large number of potential recipients. Similarly, the European Commission launched a registry of pluripotent stem cells (hPSCreg) in 2007,^15^ which has been improved in cooperation with various global organizations, such as GAiT, to establish a virtual bank of clinical-grade iPSCs.^26^ This simplifies and speeds up access for researchers and clinicians from different countries, not only reducing the cost of the technology itself but also to minimize the immune rejection problem and facilitates the possibility of bringing the iPSCs therapy to reality. Our iPSC lines are currently undergoing certification by hPSCreg, ensuring the safety, quality, and ethical aspects of the obtained cells. Furthermore, our analyses indicate that these cells exhibit low levels of HLA-DR expression, and characterizing other histocompatibility molecules will aid in determining the haplotypes of these cells, simplifying the patient-donor matching for future clinical use.

In Brazil, advanced therapy products (ATPs) require approval from ANVISA (National Health Surveillance Agency) for their use and commercialization, and significant progress has been made in the last decade. With the first Brazilian regulatory norms established in 2018 and their most recent update in 2021, 5 gene therapy products have already been approved for clinical use in the country, and there are ongoing clinical trials for the registration of new therapies.^27^ Indeed, different strategies to improve the understanding of the regulatory aspects of ATPs are being carried out,^27^ and the technology of iPSCs will accompany these advances. While the translation into clinical practice still involves a long road ahead, public and private health institutions across the country are preparing to ensure patients have access to these new products with high quality and safety. The major challenges for developing countries are the dependence on expensive imported products for the development of ATPs, as well as specialized laboratories to characterize these cells. Ensuring greater accessibility to the necessary supplies to produce iPSCs and national laboratories capable of accessing the pluripotency and safety of the cells will facilitate the advancement of this technology toward clinical application.

In our institution, besides possessing the necessary GMP and biosafety certifications required for the production of clinical-grade iPSCs, we have implemented a comprehensive production line with well-defined processes and standard operating procedures. Our experience during the validation process has enabled us to establish protocols for the induction, expansion, and creation of a master cell bank with cells that have been characterized and tested for safety for future clinical use. Some of our key insights include the recommendation that iPSCs should be expanded to at least passage 15 to minimize the possibility of detecting residual vectors. This precaution helps in reducing production costs by eliminating the need for repetitive testing. Additionally, creating a master cell bank with approximately 25 cryotubes (2 × 10^6^ cells each) not only fulfills the demand for initial analyses but also provides a stock for the establishment of working banks. Furthermore, in accordance with our legislation and GAiT’s guidelines, we recommend that this master cell bank undergoes comprehensive characterization, including identity verification (STR), assessment of sterility, endotoxins, mycoplasma, phenotyping through flow cytometry, and examination of the karyotype. These tests ensure that we can issue certificates of analysis for each master cell bank created, attesting to its quality and suitability for clinical applications.

The clinical potential of iPSC is unquestionable but standardization of reprogramming protocols and implementation of good manufacturing procedures for the manipulation of these cells are crucial to obtain safe translational products.^28^ We were able to develop the first described cGMP-compliant iPSC production pipeline for possible clinical application in the country, using commercially available and chemically defined xeno-free products.

Once the iPSC lines have been certified by hPSCreg, our roadmap is to make this service available to companies and universities worldwide that are interested in using iPSCs for basic and preclinical research. Additionally, we aim to optimize IPSC-derived differentiation protocols in specific lineages of interest to work toward precision medicine. These initial strategies will enable us to refine the services offered by the company, gaining experience, and building skilled teams. As clinical trials progress globally, we intend to seek licensing from regulatory agencies and accreditation for this service to offer it to customers who choose patient-specific iPSCs production in the future. This contribution will allow the country to play a crucial role in advancing significant developments in scientific translational medicine.

## Supplementary Material

szae010_suppl_Supplementary_Tables_1

szae010_suppl_Supplementary_Tables_2

szae010_suppl_Supplementary_Figure_1

szae010_suppl_Supplementary_Figure_2

szae010_suppl_Supplementary_Figures_Legend

## Data Availability

The data underlying this article will be shared on reasonable request to the corresponding author.

## References

[1] Yamanaka S. Pluripotent stem cell-based cell therapy—promise and challenges. Cell Stem Cell. 2020;27(4):523-531. 10.1016/j.stem.2020.09.014 33007237

[2] Guhr A , Kobold S, Seltmann S, et al. Recent trends in research with human pluripotent stem cells: impact of research and use of cell lines in experimental research and clinical trials. Stem Cell Rep. 2018;11(2):485-496. 10.1016/j.stemcr.2018.06.012 PMC6092712 30033087

[3] Kobold S , Guhr A, Mah N, et al. A Manually curated database on clinical studies involving cell products derived from human pluripotent stem cells. Stem Cell Rep. 2020;15(2):546-555. 10.1016/j.stemcr.2020.06.014 PMC7419703 32679065

[4] Dashnau JL , Xue Q, Nelson M, et al. A risk-based approach for cell line development, manufacturing and characterization of genetically engineered, induced pluripotent stem cell–derived allogeneic cell therapies. Cytotherapy. 2023;25(1):1-13. 10.1016/j.jcyt.2022.08.001 36109321

[5] Polanco A , Kuang B, Yoon S. Bioprocess technologies that preserve the quality of iPSCs. Trends Biotechnol. 2020;38(10):1128-1140. 10.1016/j.tibtech.2020.03.006 32941792

[6] Sullivan S , Stacey GN, Akazawa C, et al. Quality control guidelines for clinical-grade human induced pluripotent stem cell lines. Regen Med. 2018;13(7):859-866. 10.2217/rme-2018-0095 30205750

[7] Abberton KM , McDonald TL, Diviney M, et al. Identification and re-consent of existing cord blood donors for creation of induced pluripotent stem cell lines for potential clinical applications. Stem Cells Transl Med. 2022;11(10):1052-1060. 10.1093/stcltm/szac060 36073721 PMC9585951

[8] Álvarez-Palomo B , Veiga A, Raya A, et al. Public cord blood banks as a source of starting material for clinical grade HLA-homozygous induced pluripotent stem cells. Stem Cell Res Ther. 2022;13(1):408. 10.1186/s13287-022-02961-6 35962457 PMC9372949

[9] Tian P , Elefanty A, Stanley EG, et al. Creation of GMP-Compliant iPSCs from banked umbilical cord blood. Front Cell Dev Biol. 2022;10:835321. 10.3389/fcell.2022.835321 35372371 PMC8967326

[10] Fujioka T , Shimizu N, Yoshino K, Miyoshi H, Nakamura Y. Establishment of induced pluripotent stem cells from human neonatal tissues. Hum Cell. 2010;23(3):113-118. 10.1111/j.1749-0774.2010.00091.x 20973836

[11] Mandenius CF , Ross J. Cell-based assays using differentiated human induced pluripotent cells. In: Cell-Based Assays Using iPSCs for Drug Development and Testing. Humana, New York, NY. 2019. 10.1007/978-1-4939-9477-9_1. 31124100

[12] Nicholson NW , Ting CY, Chan DZH, Cheng YC, et al. Utility of iPSC-Derived Cells for Disease Modeling, Drug Development, and Cell Therapy. Cells. 2022;11(11):1853. 10.3390/cells11111853 35681550 PMC9180434

[13] Brasil, Agência Nacional de Vigilância Sanitária. RDC 508/2021. Dispõe sobre as Boas Práticas em Células Humanas para Uso Terapêutico e pesquisa clínica, e dá outras providências. [Accessed December 04, 2023]. https://in.gov.br/en/web/dou/-/resolucao-rdc-n-508-de-27-de-maio-de-2021-323013606

[14] Dominici M , Le Blanc K, Mueller I, et al. Minimal criteria for defining multipotent mesenchymal stromal cells. The International Society for Cellular Therapy position statement. Cytotherapy. 2006;8(4):315-317. 10.1080/14653240600855905 16923606

[15] Seltmann S , Lekschas F, Müller R, et al. hPSCreg-the human pluripotent stem cell registry. Nucleic Acids Res. 2016;44(D1): D757-D763. 10.1093/nar/gkv963 26400179 PMC4702942

[16] MacArthur CC , Pradhan S, Wetton N, et al. Generation and comprehensive characterization of induced pluripotent stem cells for translational research. Regen Med. 2019;14(6):505-524. 10.2217/rme-2018-0148 31115261

[17] Takahashi K , Yamanaka S. Induction of pluripotent stem cells from mouse embryonic and adult fibroblast cultures by defined factors. Cell. 2006;126(4):663-676. 10.1016/j.cell.2006.07.024 16904174

[18] Mandai M , Watanabe A, Kurimoto Y, et al. Autologous induced stem-cell–derived retinal cells for macular degeneration. N Engl J Med. 2017;376(11):1038-1046. 10.1056/nejmoa1608368 28296613

[19] Sugimoto N , Kanda J, Nakamura S, et al. iPLAT1: the first-in-human clinical trial of iPSC-derived platelets as a phase 1 autologous transfusion study. Blood. 2022;140(22):2398-2402. 10.1182/blood.2022017296 36112961

[20] Bloor AJC , Patel A, Griffin JE, et al. Production, safety and efficacy of iPSC-derived mesenchymal stromal cells in acute steroid-resistant graft versus host disease: a phase I, multicenter, open-label, dose-escalation study. Nat Med. 2020;26(11):1720-1725. 10.1038/s41591-020-1050-x 32929265

[21] Ramzy A , Thompson DM, Ward-Hartstonge KA, et al. Implanted pluripotent stem-cell-derived pancreatic endoderm cells secrete glucose-responsive C-peptide in patients with type 1 diabetes. Cell Stem Cell. 2021;28(12):2047-2061.e5. 10.1016/j.stem.2021.10.003 34861146

[22] Shapiro AMJ , Thompson D, Donner TW, et al. Insulin expression and C-peptide in type 1 diabetes subjects implanted with stem cell-derived pancreatic endoderm cells in an encapsulation device. Cell Rep Med. 2021;2(12):100466. 10.1016/j.xcrm.2021.100466 35028608 PMC8714853

[23] Ogliari KS , Loth FB, Halon ML, et al. Relocating to a new facility: the challenge of a cord blood banking transferral in Brazil. Transfusion. 2022;62(11):2297-2303. 10.1111/trf.17112 36250474

[24] Poetsch MS , Strano A, Guan K. Human induced pluripotent stem cells: from cell origin, genomic stability, and epigenetic memory to translational medicine. Stem Cells. 2022;40(6):546-555. 10.1093/stmcls/sxac020 35291013 PMC9216482

[25] Turinetto V , Orlando L, Giachino C. Induced pluripotent stem cells: advances in the quest for genetic stability during reprogramming process. Int J Mol Sci. 2017;18(9):1952. 10.3390/ijms18091952 28902128 PMC5618601

[26] Sullivan S , Fairchild PJ, Marsh SGE, et al. Haplobanking induced pluripotent stem cells for clinical use. Stem Cell Res. 2020;49:102035. 10.1016/j.scr.2020.102035 33221677

[27] Junior JBS. Advanced therapy products in Brazil: regulatory aspects. In: Galli MC. eds. Regulatory Aspects of Gene Therapy and Cell Therapy Products: A Global Perspective. Springer Cham; 2023:117-133. 10.1007/978-3-031-34567-8_7 37526845

[28] Ortuño-Costela MDC , Cerrada V, García-López M, Gallardo ME. The challenge of bringing iPSCs to the patient. Int J Mol Sci. 2019;20(24):6305. 10.3390/ijms20246305 31847153 PMC6940848

