# Supplementary material for: Manufacturing Parameters for the Creation of Clinical-Grade Human-Induced Pluripotent Stem Cell Lines From Umbilical Cord Mesenchymal Stromal Cells
Source: Stem Cells Transl Med. 2024 Feb 25;13(5):454–61. doi: 10.1093/stcltm/szae010 (PMC11092272; doi:10.1093/stcltm/szae010)
Supplement: szae010_suppl_Supplementary_Tables_1 [file szae010_suppl_supplementary_tables_1.docx]

| **Supplementary table 1. Outsourced laboratories and techniques used to characterize clinical grade iPSCs.** | | | | |
| --- | --- | --- | --- | --- |
| **ATTRIBUTE** |  | **TEST** | **ANALYTICAL METHOD** | **OUTSOURCED LABORATORY** |
| **Identity** |  | STR | ABI 3130 Genetic Analyzer - software Genemapper® 4 | Biospot |
| **Microbiological sterility** |  | Mycoplasma spp. | qPCR | Crop Biolabs |
|  |  | Sterility | Brazilian Pharmacopoeia (5.5.3.2) |  |
|  |  | Endotoxin | Gel Clot LAL |  |
| **Genetic fidelity & stability** |  | Karyotype | GTG Banding | Pontifical Catholic University of Paraná (PUC/PR) |
|  |  | Residual vector testing | RT-PCR | Crop Biolabs |
|  |  | SNP array | Illumina - Infinium Global Screening Array-24 (GSA)v3  BeadChip Kit (Catalog n° 20030770) | Diagnose Laboratory |
|  |  | Cancer Associated Panels | Illumina Next-Generation Sequencing (NGS) technology | Diagnose Laboratory |
| **Viability** |  | Cell Viability | CytoSmart Cell Counting | In-house |
| **Characterization** |  | Immunophenotyping | Flow cytometry | Pontifical Catholic University of Paraná (PUC/PR) |
|  |  | Gene expression (pluripotency markers) | Droplet Digital Polymerase Chain Reaction (ddPCR) | Crop Biolabs |
| **Potency** |  | Phenotypic | Embryoid body (EB) differentiation | In-house |
|  |  | Protocol: iPSCs were plated in round bottom ultra-low attachment microplate at a density of 2x104 cells/well and embryoid bodies (EB) were cultured in EB medium (Table 1) for 14 days, with the medium was changed every two days. For immunohistochemistry staining, EB were fixed with 4% paraformaldehyde for 15 min at room temperature, permeabilized with 0.3% Triton X-100 for 15 min followed by incubation with blocking solution (PBS + 10% donkey serum) for 1 h under refrigeration. Incubation with the primary antibodies (Human Three Germ Layer 3-Color Immunocytochemistry Kit – R&D Systems) was carried out overnight at 4 °C. EBs were washed and incubated with the secondary antibodies for 1 h at room temperature. Images were captured in a fluorescence microscope (EVOS imaging systems). | | |
