# Supplementary material for: Manufacturing Parameters for the Creation of Clinical-Grade Human-Induced Pluripotent Stem Cell Lines From Umbilical Cord Mesenchymal Stromal Cells
Source: Stem Cells Transl Med. 2024 Feb 25;13(5):454–61. doi: 10.1093/stcltm/szae010 (PMC11092272; doi:10.1093/stcltm/szae010)
Supplement: szae010_suppl_Supplementary_Tables_2 [file szae010_suppl_supplementary_tables_2.docx]

| **Supplementary table 2. Expanded hereditary cancer panel (264 genes)** | | | | | | | | | | | | | | | |
| --- | --- | --- | --- | --- | --- | --- | --- | --- | --- | --- | --- | --- | --- | --- | --- |
| ACD | AIP | AKT1 | ALK | ANKRD26 | APC | ARMC5 | ASCL1 | ASXL1 | ATM | ATP4A | ATR | AXIN2 | BAP1 | BARD1 | BDNF |
| BLM | BMPR1A | BPGM | BRAF | BRCA1 | BRCA2 | BRIP1 | BUB1B | CABLES1 | CASP10 | CASP9 | CBL | CD70 | CDC73 | CDH1 | CDH23 |
| CDK12 | CDK4 | CDKN1B | CDKN1C | CDKN2A | CEBPA | CEP57 | CHEK1 | CHEK2 | CREBBP | CSF3R | CTC1 | CTNNA1 | CTNNB1 | CTR9 | CYLD |
| DDB2 | DDX41 | DICER1 | DIS3L2 | DKC1 | DLST | DNAJC21 | DNMT3B | DOCK8 | EDN3 | EFL1 | EGFR | EGLN1 | EGLN2 | EPAS1 | EPCAM |
| ERCC2 | ERCC3 | ERCC4 | ERCC5 | ERCC6 | ERCC6L2 | ETV6 | EXT1 | EXT2 | EZH2 | FAN1 | FANCA | FANCB | FANCC | FANCD2 | FANCE |
| FANCF | FANCG | FANCI | FANCL | FANCM | FAS | FASLG | FBXW7 | FGFR1 | FH | FIBP | FLCN | FOXE1 | G6PC1 | GALNT12 | GATA1 |
| GATA2 | GLMN | GNAS | GPC3 | HCLS1 | HIF3A | HNF1A | HNF1B | HOXB13 | HRAS | IPMK | JAG1 | JAK2 | KDM1A | KDM3B | KIF1B |
| KIT | KLLN | KRAS | LAPTM5 | LDAH | LIG4 | LZTR1 | MAD2L2 | MAGT1 | MAP2K1 | MAP2K2 | MAP3K1 | MAX | MBD4 | MCM4 | MDH2 |
| MEN1 | MET | MITF | MLH1 | MLH3 | MMP1 | MNX1 | MRE11 | MSH2 | MSH3 | MSH6 | MSR1 | MTAP | MUTYH | MYCN | NBN |
| NF1 | NF2 | NHP2 | NME1 | NOP10 | NRAS | NSD1 | NTHL1 | NTRK1 | NYNRIN | OS9 | PALB2 | PARN | PAX5 | PBRM1 | PDGFB |
| PDGFRA | PDGFRB | PHOX2B | PIK3CA | PMS2 | POLD1 | POLE | POLH | POT1 | PPP2R2A | PPP2R3B | PRF1 | PRKAR1A | PSMC3IP | PTCH1 | PTCH2 |
| PTEN | PTPN11 | RABL3 | RAD50 | RAD51 | RAD51B | RAD51C | RAD51D | RAD54L | RAF1 | RASA2 | RASAL1 | RB1 | RBBP6 | RECQL | RECQL4 |
| RET | RFWD3 | RHBDF2 | RMI2 | RNASEL | RNF139 | RNF43 | RPS20 | RRAS | RSPO1 | RTEL1 | RUNX1 | SAMD9 | SAMD9L | SASH1 | SBDS |
| SDHA | SDHAF2 | SDHB | SDHC | SDHD | SEC23B | SETBP1 | SH2B3 | SH2D1A | SHOC2 | SLC25A11 | SLX4 | SMAD4 | SMARCA4 | SMARCAD1 | SMARCB1 |
| SMARCE1 | SOS1 | SPRTN | SRP54 | SRP72 | STAT3 | STK11 | SUFU | TERC | TERF2IP | TERT | TET2 | TEX15 | TGFBR2 | THSD1 | TINF2 |
| TMC6 | TMC8 | TMEM127 | TOP3A | TP53 | TPCN2 | TRIM28 | TRIP13 | TSC1 | TSC2 | UBE2T | USP8 | VHL | WAS | WIPF1 | WRAP53 |
| WRN | WT1 | WWOX | XIAP | XPA | XPC | XRCC2 | ZNF687 |  |  |  |  |  |  |  |  |
