# Supplementary figures and images for: Manufacturing Parameters for the Creation of Clinical-Grade Human-Induced Pluripotent Stem Cell Lines From Umbilical Cord Mesenchymal Stromal Cells
Source: Stem Cells Transl Med. 2024 Feb 25;13(5):454–61. doi: 10.1093/stcltm/szae010 (PMC11092272; doi:10.1093/stcltm/szae010)

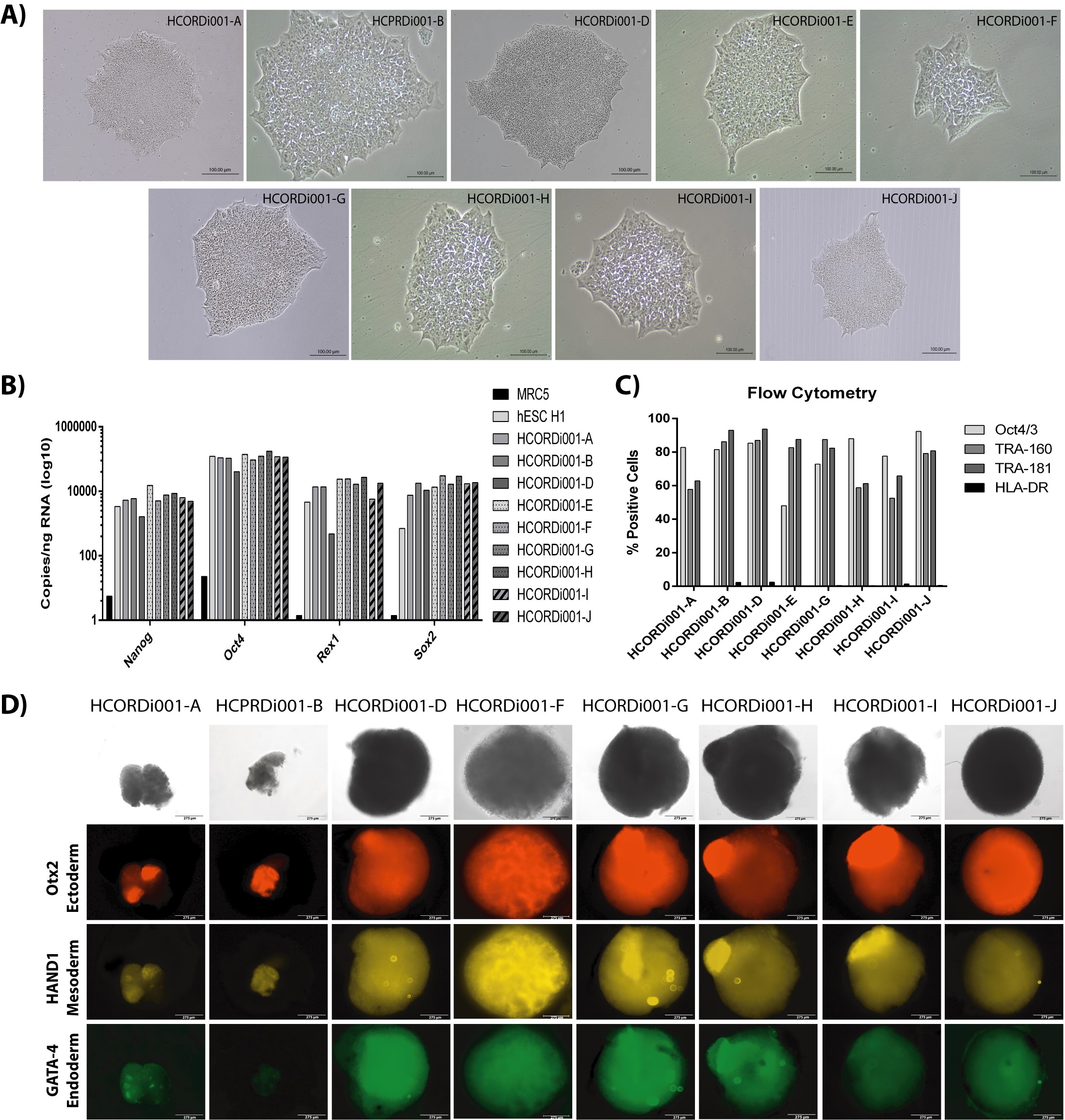

Supplement: szae010_suppl_Supplementary_Figure_1 [file szae010_suppl_supplementary_figure_1.jpeg]

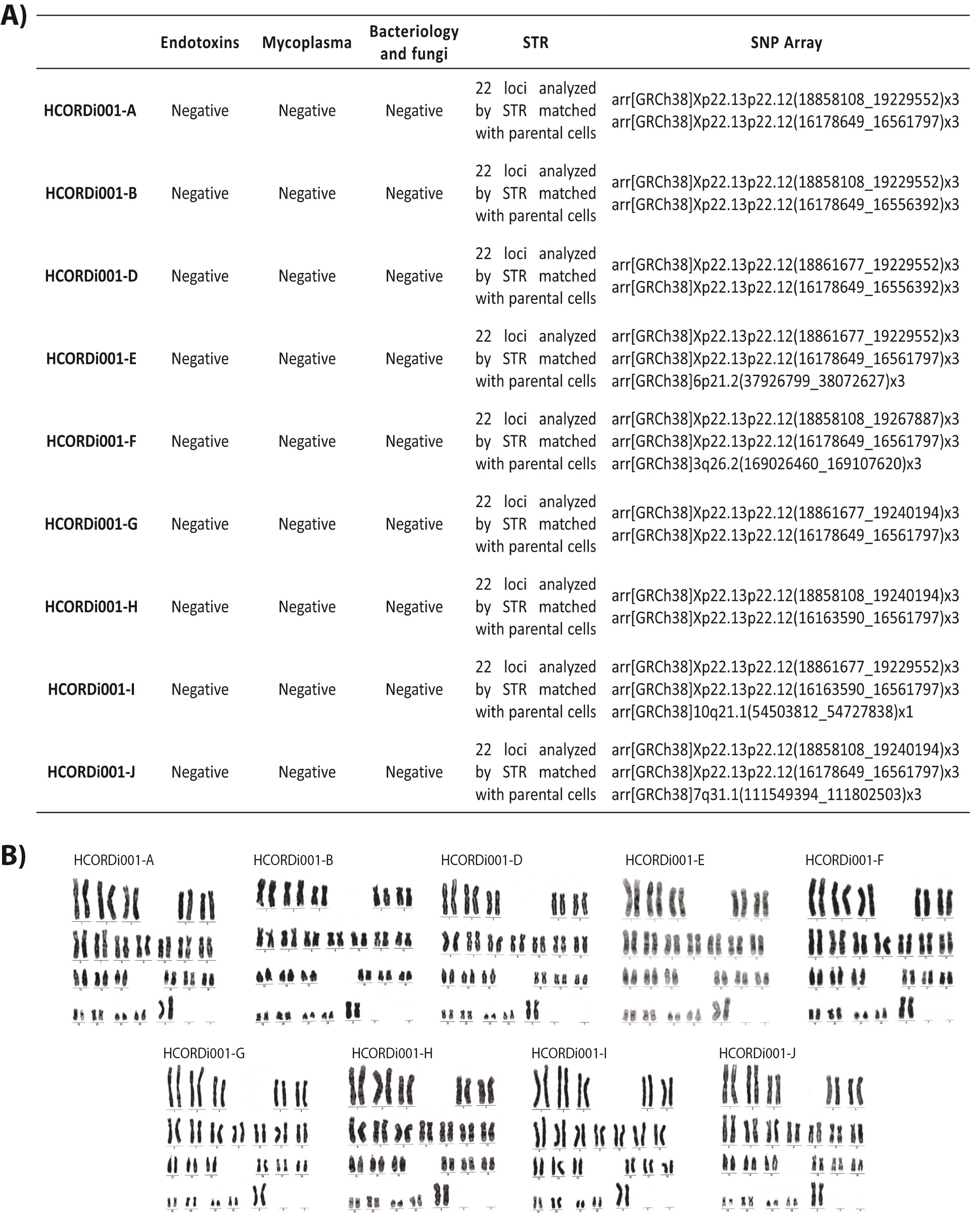

Supplement: szae010_suppl_Supplementary_Figure_2 [file szae010_suppl_supplementary_figure_2.jpeg]
