# Supplementary material for: Manufacturing Parameters for the Creation of Clinical-Grade Human-Induced Pluripotent Stem Cell Lines From Umbilical Cord Mesenchymal Stromal Cells
Source: Stem Cells Transl Med. 2024 Feb 25;13(5):454–61. doi: 10.1093/stcltm/szae010 (PMC11092272; doi:10.1093/stcltm/szae010)
Supplement: szae010_suppl_Supplementary_Figures_Legend [file szae010_suppl_supplementary_figures_legend.docx]

**Supplementary figure 1: Characterization of the other iPSC lines: (A)** Morphology of iPSC colonies between passages 10 and 16. (Scale bar: 100 μm). **(B)** Absolute quantification of pluripotency gene expression by droplet PCR digital (ddPCR). MRC5 (fibroblasts) as negative control and hESC H1 (embryonic cells) as positive control**. (C)** Stemness markers used to evaluate iPSC by flow cytometry. The HCORDi001-F iPSC line did not have its result released by the outsourced laboratory due to technical problems in the analysis and will be re-analyzed. **(D)** Immunofluorescent staining for Ectoderm marker Otx2, mesoderm HAND1 and Endoderm GATA-4. Scale bar: 275μm. The HCORDi001-E iPSC line did not have its result released due to technical problems in the analysis and will be re-analyzed.

**Supplementary figure 2: Safety assessment of the other iPSC lines: (A)** Panel of sterility analyses (endotoxins, mycoplasma and bacteria/fungi), STR and variants detected in the SNP array. **(B)** All iPSCs showed a normal karyotype after culture at least until passage 10.
